# A multi-country, prospective cohort study to evaluate the economic implications of relapse among children recovered from severe acute malnutrition: a study protocol

**DOI:** 10.1186/s40795-022-00631-7

**Published:** 2022-11-26

**Authors:** Chloe Puett, Sarah King, Heather Stobaugh

**Affiliations:** 1 Department of Family, Population & Preventive Medicine, Program in Public Health, Health Sciences Center, Stony Brook University, Stony Brook, NY USA; 2 Action Against Hunger, New York, NY USA; 3 Friedman School of Nutrition Science and Policy, Tufts University, Boston, MA USA

**Keywords:** Community-based management of acute malnutrition, Relapse, Severe acute malnutrition, Cost-efficiency analysis, Institutional costs, Wasting; kwashiorkor, Marasmus, Post-discharge outcome

## Abstract

**Background:**

Community-based management of acute malnutrition (CMAM) is an effective intervention at recovering children from severe acute malnutrition (SAM) and preventing mortality. However, there is growing evidence that for many children recovery is not sustained post-discharge. This study will assess the economic implications of relapse by calculating the average cost of treating a case of SAM that relapses after initial CMAM treatment compared to the cost of a case that remains recovered for 6 months post-discharge.

**Methods:**

This protocol outlines the methods for a cost-efficiency analysis to assess cost per episode of treatment for acute malnutrition for children enrolled in CMAM programs for initial SAM treatment in Mali, Somalia and South Sudan. Cost data will be collected and analyzed on a monthly basis for each CMAM service component (outpatient treatment program for SAM, supplementary feeding program for moderate acute malnutrition, and inpatient stabilization care for SAM with medical complications). Financial data will be extracted from expenditure records from institutional accounting systems where possible. Where these are not present, cost data will be collected via interview and review of financial documents. Staff time allocation interviews will be conducted. This data will be applied to quantify personnel costs, to apportion costs that are shared between programs and to exclude staff time spent on research activities.

**Discussion:**

This study will provide the first estimates to address the limited evidence on the economic implications of SAM relapse in CMAM programs. Data from this economic analysis will help raise awareness and provide actionable data for the global nutrition community to address the financial burden of relapse. Estimating the cost of relapse in three countries representing different geographic and operational contexts will help in generalizing these results.

**Trial registration:**

Registration # IORG0007116, Date of registration: 06/09/2020. This study is not registered as a clinical trial as it is observational research and does not include an intervention. The study has received the required ethical approvals as outlined in the declarations.

**Supplementary Information:**

The online version contains supplementary material available at 10.1186/s40795-022-00631-7.

## Background

Over the past 20 years, developments in the community-based management of acute malnutrition (CMAM) model have improved outcomes in acutely malnourished children globally [1–3]. While CMAM programs are effective at recovering children, there is growing evidence that recovery may not be sustained well after discharge from programs [4–11]. Further, post-discharge outcomes are not measured in a standardized way which limits understanding, analysis and remediation of this challenge [12].

Notwithstanding their effectiveness in reducing mortality outcomes, CMAM programs also have been found to be a resource-intensive intervention relative to other intervention options for addressing child undernutrition [13]. It is therefore important from an economic perspective to ensure efficient use of these resources. Assessing use of resources allocated for re-treating children who have relapsed to acute malnutrition (AM) will help raise awareness within the global nutrition community on the financial implications of relapse and the potential need to adapt current programming to reduce relapse rates and improve cost-efficiency.

This document defines the protocols and indicators to be used in collecting and analyzing financial data for three CMAM programs in Mali, Somalia and South Sudan, which are included in a parent study to assess relapse in young children after treatment for severe acute malnutrition (SAM) and its costs. The parent study is hereafter called the “SAM Relapse Study”. Within this overarching study, an economic analysis will be conducted (hereafter called the “costing sub-study”) in which the aim is to assess the financial costs of re-treating children who experience relapse to SAM within six months of initial recovery from SAM through CMAM programs.

## Methods

### Study design

**Fig. 1:**
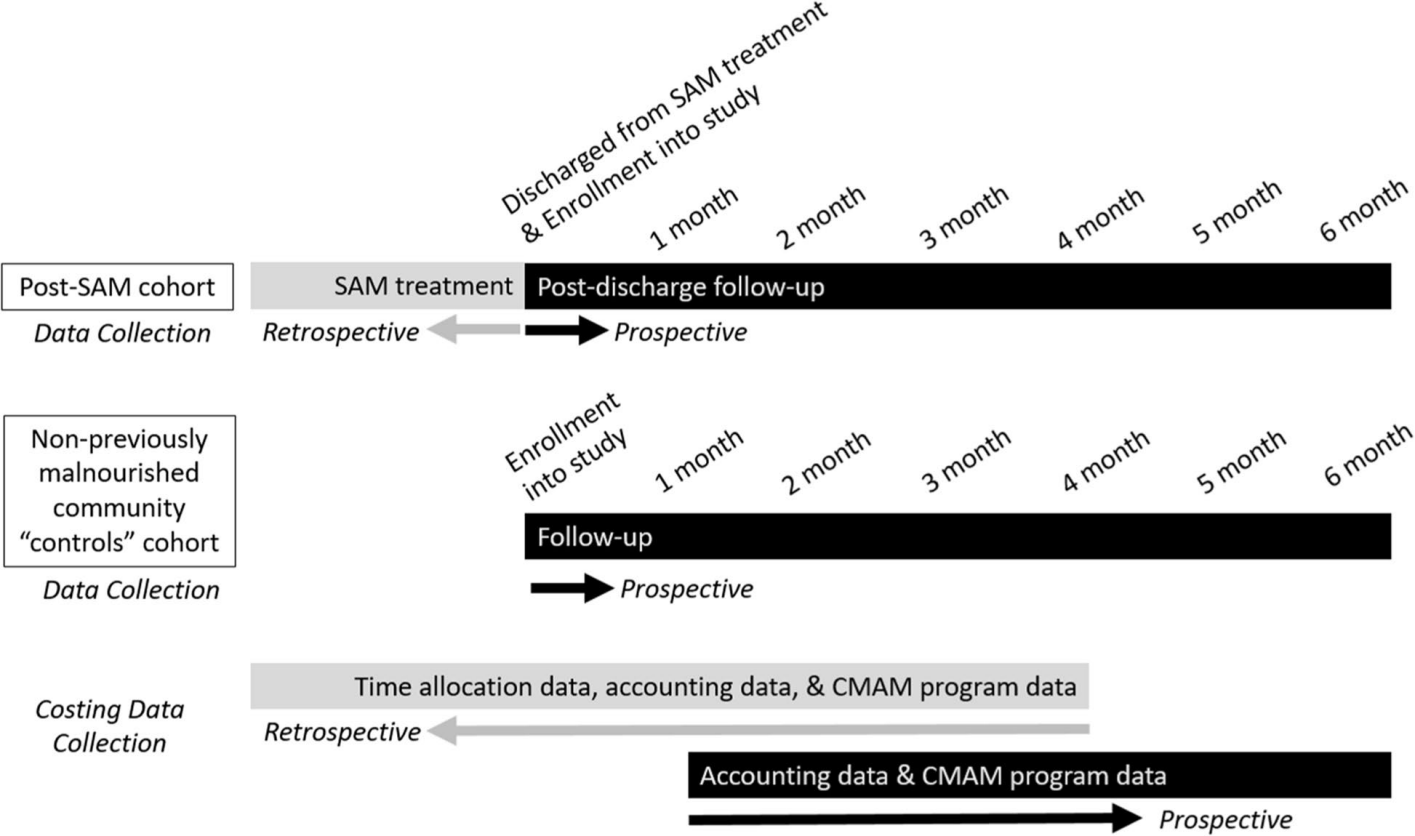
Overview of overarching SAM Relapse Study design and costing sub-study data collection timing

This article outlines the methodology for this costing sub-study within the larger overarching SAM Relapse Study. The study protocol for the overarching SAM Relapse Study will be submitted for publication elsewhere; however, a summary of the study design is as follows. The SAM Relapse Study comprises of a prospective cohort following in parallel over 1,800 post-SAM children 6–59 months and over 1,100 matched community controls for six months in three different countries: Somalia, South Sudan, and Mali. After being discharged as recovered from SAM, post-SAM and control children are enrolled into the study and followed monthly to assess for AM, including SAM or moderate acute malnutrition (MAM) (Fig. 1). The study objectives for the parent study are to: (1) compare the cumulative incidence of AM among children after recovery from SAM in outpatient treatment programs with the cumulative incidence of AM among children who did not previously experience SAM; (3) identify child- and household-level factors associated with relapse; and (4) to infer possible CMAM programmatic factors that may influence relapse when comparing relapse rates across the three different programs and contexts.

Cost data will be collected for ongoing CMAM programs in each country. The standard CMAM model includes four service components: 1) inpatient treatment for SAM with medical complications in stabilization centers (SC); 2) outpatient treatment for SAM without medical complications through outpatient therapeutic programs (OTP); 3) outpatient care for MAM in supplementary feeding programs (SFP); and 4) community outreach services to include active case finding/screening, community mobilization, and sensitization activities.

While all countries included in the analysis provide each of the four service components, there are slight variations in implementation (Table 1). For example, the management of uncomplicated SAM cases in Somalia and South Sudan begins with treatment in OTP until a child progresses to the MAM phase of AM at which point the child is transferred to the SFP program to complete treatment. Whereas, in Mali, the program treats a child initially presenting with SAM in OTP until full recovery with no transfer. As such, children receiving care in Mali will receive more frequent follow-up visits and only one type of therapeutic product until recovery during initial treatment. Conversely, children in South Sudan and Somalia will receive both RUTF and RUSF and varied follow-up through treatment given the transfers between service components. These differences will be accounted for in the collection and analysis of costing data. Nonetheless, it is not anticipated that these slight differences will strongly bias study results.

**Table 1:** CMAM service provider and overview of point of care service components for children admitted for treatment by country

**Country**	**Primary Implementer**	**Outpatient Therapeutic Program (OTP)**	**Supplementary Feeding Program (SFP)**	**Stabilization Center (SC)**
Mali	Ministry of Health (MoH)	- Treat SAM children until full recovery from AM (including through the MAM phase) - Weekly follow-up consultation visits at point of care - RUTF provided	- Treat children who initially present as MAM until full recovery - Bi-weekly follow-up consultation visits at point of care - RUSF provided	- Treat SAM children with medical complications - Around the clock inpatient care provided until child stabilizes and can enter OTP - F75 and F100 provided for acute and transition phases respectively
Somalia	Combined (MoH + Action Against Hunger)	- Treat SAM children until children transition from the SAM to MAM phase of treatment, at which point they are referred to SFP for the remainder of treatment - Weekly follow-up consultation visits at point of care - RUTF provided	- Treat children referred from OTP and those initially presenting as MAM through full recovery of MAM - Bi-weekly follow-up consultation visits at point of care - RUSF provided	
South Sudan	Action Against Hunger	- Treat SAM children until children transition from the SAM to MAM phase of treatment, at which point they are referred to SFP for the remainder of treatment - Weekly follow-up consultation visits at point of care - RUTF provided	- Treat children referred from OTP and those initially presenting as MAM through full recovery of MAM - Bi-weekly follow-up consultation visits at point of care - RUSF and corn-soya blend provided	

*Abbreviations*: *AM* Acute malnutrition, *CMAM* Community-based management of acute malnutrition, *MAM* Moderate acute malnutrition, *MoH* Ministry of Health, *OTP* Outpatient therapeutic program, *RUSF* Ready-to-use supplementary food, *RUTF* Ready-to-use therapeutic food, *SAM* Severe acute malnutrition, *SC* Stabilization center, *SFP* Supplementary feeding program

### Costing sub-study aim

The overarching aim of the costing sub-study includes calculation of unit costs for different CMAM service components, and a cost-efficiency analysis using these unit costs to assess the financial burden of re-treating children who experience relapse after full recovery from initial SAM treatment in CMAM programs. Cost-efficiency analysis enables examination of average costs incurred to produce an output. The output in this case is a treatment episode for a case of AM, both during initial treatment and during instances of re-treatment for relapse. The cost data collection and analysis procedures described below follow recommended processes outlined in costing guides for CMAM programming [14, 15].

Assessing the average costs per treatment episode will enable an understanding of the average costs of a relapse compared to the average cost per sustained recovery. Cost-effectiveness will not be assessed as the overarching SAM Relapse Study is not measuring the effectiveness of an intervention.

Data collection and analysis will be completed in four steps: 1. assembling total programmatic costs for CMAM service components (e.g., OTP, SFP, SC); 2. adjusting costs to include only relevant costs (i.e., excluding research and non-CMAM activities); 3. calculating average costs per child receiving services in the CMAM program; and 4. analyzing those costs as they relate to retreatment for relapse episodes.

### Costs to be collected

Financial data regarding all institutional-level costs incurred with implementing the CMAM programs in each country will be collected throughout the study period. Institutional-level costs are those costs associated with direct implementation as incurred by the institution implementing services. Institutional costs do not include societal costs, such as time spent by caregivers/children or opportunity costs incurred by the community. Societal costs will not be collected given the burden to caregivers required to collect such costs and the lack of direct implication for the purpose of this study. All costs related to research-specific tasks will be excluded from this analysis as these do not affect the quality or process of implementation, and as the purpose of the analysis is to estimate costs of program implementation under typical, non-research settings.

Institutional-level costs associated with the CMAM program that will be collected in this study include staffing, direct implementation, transportation, office running, and capital costs (Table 2). Regarding staffing, costs will be collected for all staff whose tasks and responsibilities have direct implications on CMAM service provision, from site-specific staff up to the level of country director for implementing NGOs and clinic staff for Ministries of Health. Since not all staff provide full-time support to CMAM operations, staff costs will be split according to staff time allocation in order to quantify personnel costs directly for CMAM program implementation, management, and support. Time allocation data will also be used to apportion costs that are shared between CMAM-related activities and non-CMAM activities to exclude staff time spent on activities not relevant to this analysis.

**Table 2:** Institutional-level costs associated with CMAM program implementation for which financial data is to be collected

**Category**	**Individual Elements**
Staffing Costs	Program management (implementation level) Clinic-based staff (implementation level) Support staff (implementation/national level) Coordination staff (capital/national level)
Direct Implementation Costs	Supplies (non-food/non-medicine) Food commodities (e.g., RUSF, RUTF, CSB + +) Medicines (e.g., amoxicillin) Facility running costs (e.g., rent, security) Facility construction/repair Trainings
Transportation Costs	Freight of supplies, commodities & equipment (international and domestic) Warehouse & handling costs Vehicle Running costs
Office Running Costs	Office running costs (e.g., generator, utilities, security, rent) Office supply costs (e.g., stationary, equipment) Other direct costs (e.g., phones, phone credit)
Capital Costs	Vehicles Other assets (e.g., computers)

Regarding direct implementation, costs will focus specifically on those non-staff inputs, infrastructure, and activities involved in direct service provision of AM treatment as well as those costs supporting treatment access (e.g., active community screening, community mobilization efforts). Excluded costs will include the cost for treatment of malnourished pregnant and lactating women, as this population does not meet the enrollment criteria for the larger SAM Relapse Study. Also excluded will be costs for preventive nutrition activities, wider nutrition assessments, and surveys.

Food commodities are a critical input in CMAM programs. Yet, the associated supply chain costs are notoriously difficult to capture, particularly those incurred prior to reception from the CMAM service provider. In order to capture an accurate quantity purchased and distributed, two approaches will be implemented. First, the total costs from food commodity waybills will be apportioned across facilities according to caseload at each facility. Only costs associated with those facilities included in the SAM Relapse study will then be included in the final analysis. This approach ensures all costs associated with the total amount of food procured are incorporated, including wastage (e.g., spoilage, theft, loss). However, this approach does not reflect the exact cost of products distributed to each child. Therefore, a second approach will be used that better captures the exact amount received by each child in treatment. This involves adding the total cost of the product and all other procurement costs (e.g., transportation, storage), then dividing that total by the total number of sachets involved in that procurement. Using CMAM programmatic data, the cost per sachet will be multiplied by the number of sachets each child receives, which then will be summed across all children in the program. This second method will capture the cost of product received by each individual but will not reflect larger product loss. All calculations will be compared for validity. This dual approach will only be applied to food commodities and no other commodities because of their large share of implementation costs. For all other commodities, the total costs from waybills and accountancy records will be used.

Transportation costs will include freight and cargo costs, domestic distribution costs, and warehouse and handling costs throughout the supply chain. There will be effort given to obtain all transportation costs associated with the full spectrum of the food commodity supply chain. Additionally, transport costs associated with implementation will be captured. This can include but is not limited to transport associated with active screening and community mobilization, supportive supervision, trainings and ambulance services.

The cost of capital items will be amortized using standard tables (three years for computers and five years for all other equipment) and discounted at a rate of 3%.

### Data collection

All data will be collected through two methods: (1) review of accounting and financial records, and (2) key informant interviews. First, extracting costs from expenditure records from institutional accounting systems will be sought and completed where possible. Where accountancy data is not available, such “off-budget costs” will be collected via key informant interviews with program and finance staff. These include costs incurred by partner organizations, i.e., MoH or other NGOs, such as medical supplies and facility running costs. If possible, data provided through key informant interviews will be verified through review of other documentation, in the form of quotes, websites, reports, etc. These costs will be estimated using an off-budget “ingredients” approach where each individual component of service delivery is quantified and costed [16]. This includes, for example, taking monthly time allocation estimates from MoH staff and quantifying them using monthly salary data.

Staff time allocation interviews will be conducted by the study team in each country using data collection tools designed to capture time allocated between CMAM-specific program activities and other non-CMAM programs. Information gathered will also capture time allocated to each of the different CMAM service components (e.g., SC, OTP, SFP). This data will not include CMAM program set-up, as the programs in each of the three countries are well-established. Time allocation will be relevant for regular, ongoing routine implementation activities. Additionally, all data on staff time will be used to estimate monthly personnel costs and to apportion shared costs.

Data will be collected on an ongoing basis and compiled for the duration of the study, beginning from the start of initial treatment of the first children enrolled in the SAM Relapse study to the end of the post-discharge six-month follow-up period of the last child to participate in the study, equating to January 2021 to December 2022. It is likely, however, that a child may relapse during the latter part of the post-discharge follow-up period for which treatment will be required. That treatment may continue beyond the study timeframe, rendering data collection on those specific costs unfeasible within the confines of this study.

Accounting data will be compiled on a quarterly basis and reviewed on an ongoing basis to remove any costs not relevant to the study. All costs will be adjusted for inflation and converted to 2021 USD (or the most recent year for which inflation data is available).

### Data analysis

Indicators to be used in the analysis are described in the following sections and an indicator matrix is outlined in Table 3.

**Table 3:** Indicator matrix

**Indicator(s)**	**Definition/description**	**Data Collection Method**	**Time period**
Monthly cost per child per CMAM service component	Average monthly cost incurred per child in each service (SC, OTP, SFP). Estimated as: the total costs per service per month divided by the number of children who were enrolled in each service per month	Review of financial records, off-budget costs collected via interviews, time allocation interviews, study data, and CMAM program records	Monthly
Length of Stay (LoS) per child per CMAM service component	Average length of stay in each service, defined as the time from admission to discharge in each SC, OTP, and SFP separately. (When a child transfers from one component to another, this equates to a “discharge” and marks the end of his stay in that component of the CMAM program.) This will be estimated in months (and fractions of months) and calculated for initial SAM episode treatment, relapse to AM episode retreatment, and time in retreatment for any and all episodes of relapse to AM during the six months following initial SAM recovery	study data; and CMAM program records	Entire study period
Average cost per child for initial SAM recovery	The overall cost to recover children enrolled in treatment for initial episode of SAM. Calculated using methods comparable to standard CMAM costing studies that do not account for relapse. Estimated as: the total costs of initial SAM treatment divided by the number of children recovered in initial SAM treatment	Review of financial records, off-budget costs collected via interviews, time allocation interviews, study data, and CMAM program records	Entire study period
Average cost of initial treatment for study eligible children	The cost per initial treatment episode (only for children recovered who were eligible for inclusion in the study) to be used in estimating total per-child treatment costs. This represents the average cost of treating a child with SAM who does not relapse. Estimated as: total cost of initial SAM treatment multiplied by the proportion of children recovered in initial treatment, divided by the number of children recovered in initial SAM treatment	Review of financial records Off-budget costs collected via interview Time allocation interviews # of children enrolled per service and LoS from study records	Entire study period
Average cost of retreatment for relapses during six-month post-discharge period	The average cost of retreatment for any and all relapse(s) to AM episodes within six months post-discharge from initial recovery. Estimated as: monthly cost per child per service multiplied by the average LoS in each service (in months) and for relapsed children	Review of financial records, off-budget costs collected via interviews, time allocation interviews, study data, and CMAM program records	Entire study period
Total average cost of relapse	The average total cost incurred for treatment of a child who relapses fails to sustain recovery for at least six months post initial recovery. Estimated as: cost of initial treatment + cost of retreatment for relapses during six-month post-discharge period	Review of financial records, off-budget costs collected via interviews, time allocation interviews, study data, and CMAM program records	Entire study period

#### Monthly program costs per service component

Total program cost estimates will be compiled on a monthly basis during the study timeframe. Total costs will be apportioned to each CMAM service component (e.g., OTP, SFP, SC) according to allocations established through interviews and accountancy data to determine monthly costs associated with each service component provided.

#### Monthly cost per child per service component

Using monthly CMAM program data that details the total number of children receiving care in each of the CMAM service components, the total costs per service per month will be divided by the number of children in each service for each month. This will estimate the average monthly cost of treatment per child in each service. For example, the calculation for OTP in Month “A” will be as follows:

$$\mathrm{Month}\;\mathit{``}\text{A''}\;\mathrm{total}\;\mathrm{OTP}\;\;\mathrm{costs}\;\mathrm{per}\;\mathrm{child}\;=\;\frac{\mathrm{Total}\;\mathrm{Month}\;\mathit{``}\text{A''}\;\mathrm{OTP}\;\mathrm{Service}\;\mathrm{Costs}}{\mathrm{Total}\;\mathrm{No}.\;\mathrm{Children}\;\mathrm{in}\;\mathrm{OTP}\;\mathrm{in}\;\mathrm{Month}\;\mathit{``}\text{A''}}$$

Each of the monthly costs per child per service type will then be added across the life of the study and divided by the total number of months of program implementation during the study to determine an average monthly cost per child per service type.

#### Cost per initial SAM recovery

Using programmatic data, the average length of stay (LoS) in each of the different CMAM service components will be calculated in fractions of months for study children who are admitted for initial SAM treatment and recover. Since many children transfer across CMAM service components, the cost of each component will be added together to encompass the total CMAM services provided for the entire LoS for that episode’s treatment. For example, we will calculate that, on average, a child who recovers from an initial SAM episode spends “X” months in OTP plus “Y” months in SFP. The calculation for the cost per initial SAM recovery will be as follows:

$${{c}_{t}=\overline{c}}_{OTP}{\overline{L}}_{OTP}+{\overline{c}}_{SFP}{\overline{L}}_{SFP}$$

where C_t_ is the cost per initial SAM recovery, C_OTP_ and C_SFP_ are the average monthly costs of OTP and TSFP per child, respectively, and L_OTP_ and L_SFP_ are the average length of stay (in fractions of months) in OTP and SFP of children who recover from initial SAM treatment.

#### Cost per SAM child treated and SAM child recovered

To produce indicators comparable to other CMAM costing studies, we will also calculate unit costs for SAM treatment outcomes. These indicators will also be used as measures of program performance to compare across the three study countries. This includes the cost of children initially treated for SAM regardless of discharge outcome and the cost of those who are discharged as recovered. These will be analyzed in two ways: First, an average cost per child treated will be calculated as total number of children treated in initial SAM treatment divided by the total cost of initial SAM treatment across the duration of program implementation (Eq. 1).

1 $$\mathrm{Average}\;\mathrm{cost}\;\mathrm{per}\;\mathrm{SAM}\;\mathrm{child}\;\mathrm{treated}=\frac{\mathrm{Total}\;\mathrm{Number}\;\mathrm{of}\;\mathrm{Children}\;\mathrm{Receiving}\;\mathrm{Initial}\;\mathrm{SAM}\;\mathrm{Treatment}}{\mathrm{Total}\;\mathrm{Cost}\;\mathrm{of}\;\mathrm{Initial}\;\mathrm{SAM}\;\mathrm{Treatment}\;\mathrm{Across}\;\mathrm{Duration}\;\mathrm{of}\;\mathrm{Program}}$$

Second, the cost of only the recovered children (who were eligible for inclusion in the larger SAM Relapse Study) will be calculated as the total number of children discharged as recovered divided by the total cost of initial SAM treatment across the duration of the program implementation (Eq. 2).

2 $$\mathrm{Average}\;\mathrm{cost}\;\mathrm{per}\;\mathrm{child}\;\mathrm{recovered}=\frac{\mathrm{Total}\;\mathrm{Number}\;\mathrm{of}\;\mathrm{Children}\;\mathrm{Discharged}\;\mathrm{as}\;\mathrm{Recovered}\;\mathrm{in}\;\mathrm{Initial}\;\mathrm{SAM}\;\mathrm{Treatment}}{\mathrm{Total}\;\mathrm{Cost}\;\mathrm{of}\;\mathrm{Initial}\;\mathrm{SAM}\;\mathrm{Treatment}\;\mathrm{Across}\;\mathrm{Duration}\;\mathrm{of}\;\mathrm{Program}}$$

#### Cost of child who fails to sustain recovery

The total cost incurred by a child who fails to sustain recovery for six-months following initial SAM treatment will build upon formulas presented above and additional data from the parent SAM Relapse Study. For those who relapsed, the average monthly cost per retreatment per service type will be calculated. This will then be multiplied by the average LoS (in fractions of months) per each of the service types (e.g., OTP, SFP, and SC) throughout the entire six-month post-discharge follow-up period. Those who relapse may spend time in the SC, which will also be included in the calculation if applicable. Conversely, if a child relapses and only spends time in the SFP, then only this time will be applied. This calculation will account for all relapse episodes if and where multiple relapses occurred. The computation is as follows:

$${\overline{C}}_{r}{=\overline{c} }_{OTP} {\overline{L} }_{OTP}+{\overline{c} }_{SFP} {\overline{L}}_{SFP}+{\overline{c} }_{SC}{\overline{\mathrm{L}} }_{SC}$$

where C_r_ is the average cost of retreatment for relapse during the six-month post-discharge period, C_OTP_, C_SFP_ and C_SC_ are the average monthly costs of OTP, SFP and SC per child, respectively, and L_OTP_, L_SFP_ and L_SC_ are the average length of stay (in fractions of months) in OTP, SFP, and SC of children who require retreatment.

It is important to note that the length of stay in treatment for children who relapse will likely be shorter in this study than in a non-study setting. Because study resources only allow for a six-month post-discharge follow-up period, many of the children who relapse will likely still be receiving treatment by the end of the study (or the end of the six-month post-discharge follow-up period). Thus, the average length of stay will not necessarily account for the full time a child is treated until discharge of a relapse episode.

The cost of retreatment for relapse will then be added to the cost per child recovered from initial SAM treatment, in order to encompass the cost of treating a child for SAM and the costs of retreating the same child for relapse(s) during the subsequent six months. The calculation is:

$${\overline{C}}_{f}=\sum {\overline{C}}_{i} {\overline{C}}_{r}$$

where C_f_ (the average cost per child who fails to sustain recovery) equals the sum of the average cost per child recovered during initial SAM treatment (C_i_) and the average cost of retreatment for relapse during the six-month post-discharge period (C_r_).

Study data will be explored for the possible identification of certain patterns of relapse pathways in each country which may allow for appropriate and relevant disaggregation of results.

### Cost efficiency analysis

The unit cost estimates outlined above will enable calculation and comparison of the average cost of treating a child of SAM that relapses after initial CMAM treatment with the cost of a child with SAM that remains recovered for six months post-discharge. It will also allow for the comparison of an average cost of initial episode of SAM treatment with an average cost of relapse episode of SAM retreatment.

## Discussion

While there is a growing evidence base on the costs of treating AM [17–19], currently there is no evidence on the sustainability of these programmatic investments. Inefficiencies arise when scarce resources are used in re-treating children who relapse to AM after recovery and program discharge. This study will provide the first estimates to address the limited evidence on the economic implications of relapse after initial SAM treatment. Furthermore, at the time of this publication, this protocol is the first to propose specific indicators for estimating the cost of relapse and a process for analyzing the economic burden of relapse.

There are a few limitations associated with the design of this study. First, the follow-up time period is relatively short: only six months. When taking into perspective that the duration of treatment itself for many children could be up to six months, this limits the ability to capture fully the extent of relapse over a year or more. Second, the focus of this analysis is on institutional resource use, rather than a societal approach, which would include both institutional and societal costs. Therefore, this study will not provide information on the expenses and opportunity costs for households in repeating treatment. Furthermore, it will not capture additional household costs that may be incurred in other health seeking behaviors or other healthcare costs associated with a child failing to maintain normal nutrition status following initial treatment. Future research could explore the societal dimension of relapse costs.

Data from this economic analysis will help raise awareness and provide actionable information for the global nutrition community on the financial burden of the sustainability of SAM recovery. This may carry implications for the need to adapt current programming to improve effectiveness and reduce relapse rates and associated inefficient resource use. Estimating the cost of relapse in three programs representing different geographic and operational contexts will help in generalizing these results.

## Supplementary Information

**Additional file 1. **Interview guide.

## Data Availability

The datasets generated and analyzed during the current study are not publicly available due to their sensitive internal financial nature but may be available from the corresponding author on reasonable request and with permission of Action Against Hunger.

## Abbreviations

CMAM: Community-based management of acute malnutrition
AM: Acute malnutrition
SAM: Severe acute malnutrition
MAM: Moderate acute malnutrition
SC: Stabilization center
OTP: Outpatient therapeutic program
SFP: Supplementary feeding program
RUTF: Ready-to-use therapeutic food
RUSF: Ready-to-use supplementary food
MoH: Ministry of Health
NGO: Non-governmental organization
CSB: Corn-soy blend
USD: United States Dollars
LoS: Length of stay
