# Supplementary material for: A multi-country, prospective cohort study to evaluate the economic implications of relapse among children recovered from severe acute malnutrition: a study protocol
Source: BMC Nutr. 2022 Nov 26;8:139. doi: 10.1186/s40795-022-00631-7 (PMC9701364; doi:10.1186/s40795-022-00631-7)
Supplement: Supplementary file 1 — Additional file 1. Interview guide. [file 40795_2022_631_MOESM1_ESM.docx]

# ACF staff

This interview is a part of the cost analysis that is being conducted for the Relapse study by the Research department at Action Against Hunger across 3 country offices: Mali, Somalia and South Sudan. The purpose of the costing analysis is to estimate the average cost of treatment for acutely malnourished children, by both ACF and MoH in the 3 countries, in order to estimate the additional cost of repeating treatment for children who have relapsed. The results of the analysis will show how much more it costs to treat a relapsed case, beyond the cost of initial treatment. This information will be used to advocate for efforts to reduce relapse by improving the adherence, coverage, uptake and eventual effectiveness of initial treatment for acute malnutrition.

I am going to ask you some questions about the time that you spend on services for acute malnutrition that you are involved in as part of your work. This information is not being collected to evaluate how much you work or the quality of your work, but instead to understand how much staff time is being spent on different activities so we can apply an accurate cost of your time for the costing study.

**For implementation & management staff**

On average, how many hours per week do you work?

(Walk through usual hours per day of the week and add up)

How many days (or hours) of this time is spent on CMAM activities, including OTP and TSFP?

(Take their answer, get the corresponding % time and tell it back to them. For example, if they say they work 8 hours (1 day) per week on CMAM, confirm with them. “So, you think you spend about 20% of your time or 1 day per week on CMAM?” This may help them to refine their answer).

So, you think you spend about __% or __ day(s) per week on CMAM? (Record their final % to CMAM and non-CMAM.)

Of the time you spend specifically on CMAM, what proportion of time is spent on OTP, TSFP, and PLW services? Record their final % time to CMAM sub-activities.

For Research Staff: What proportion of your time do you spend on activities that either support the Operations team or could influence program quality, instead of just observing it? (These will be the operations support activities.)

**For support staff**

(This section is for staff who are not involved in program activities, such as Finance, HR, Logs, CD, etc.)

How many programs are currently being run at this office? (Listen to their list of programs, make a list of them and note the total number)

Do all of these programs take the same amount of time for you to support? (If “Yes”, divide 1 by the total # of programs and this is the % time per program. If “No”, revise the estimates for the various programs up or down until they are satisfied with the percentages.)

So, you think you spend about __% or __ day(s) per week on CMAM? (Record their final % to CMAM and non-CMAM in **columns B&C**. This amount of time should then be evenly split among the different CMAM sub-activities being implemented at your country office.)

# MoH Staff

This interview is a part of the cost analysis that is being conducted for the Relapse study by Action Against Hunger. The purpose of the costing analysis is to estimate the average cost of treatment for acutely malnourished children, by both ACF and MoH, in order to estimate the additional cost of repeating treatment for children who have relapsed. The results of the analysis will show how much more it costs to treat a relapsed case, beyond the cost of initial treatment. This information will be used to advocate for efforts to reduce relapse by improving the adherence, coverage, uptake and eventual effectiveness of initial treatment for acute malnutrition.

**Case management tab**

I am going to ask you some questions about your time allocation to services for acute malnutrition that you are involved in as part of your work. This information is not being collected to evaluate how much you work or the quality of your work, but instead to understand how much staff time is being spent on different activities so we can apply an accurate cost of your time for the costing study.

Which services are you involved in?

- OTP
- TSFP
- SC

OTP:

How many children are admitted to OTP per month on average? (Ask them also to try to give an average if possible.)

How many times per month are OTP sessions offered at this facility?

How much time do you spent with each child admitted to OTP? (Ask them also to try to give an average if possible.) OR if recording length of total OTP session instead of time with each child, ask “How much time does each OTP session last?”

Do you spend any additional time per month on OTP, beyond time with patients, such as for reporting or other related administrative tasks?

TSFP:

How many children are admitted to TSFP per month on average? (Ask them also to try to give an average if possible.)

How many times per month are TSFP sessions offered at this facility?

How much time do you spent with each child admitted to TSFP? (Ask them also to try to give an average if possible.) OR if recording length of total OTP session instead of time with each child, ask “How much time does each TSFP session last?”

Do you spend any additional time per month on TSFP, beyond time with patients, such as for reporting or other related administrative tasks?

SC:

How many children are admitted to SC per month on average? (Record any details on range, etc.)

What proportion of the admissions to SC are under 6 months old?

What is the average number of days spent at the facility per child admitted to SC?

How much time do you spend admitting each case to SC?

How much time, on average, do you spend managing each case at SC during the day (24-hour period)? (i.e., # of visits to their bed and time spent per visit. Ask them also to try to give an average if possible.)

How much time do you spend discharging each case from SC?

Do you spend any additional time per month on SC, beyond time with patients, such as for reporting or other related administrative tasks?

All staff:

How many hours do you work per week, on all activities at the health facility, on average? We are not trying to judge how much you work, but to get an average number so we can estimate a percent of your time allocation for CMAM.

How many days do you work per month, on average?

**Other activity time tab**:

What are some of the other activities related to acute malnutrition that you work on, on a monthly or weekly basis? (Get a list of activities per staff. For each activity, ask the following questions, walking them through their time allocation, getting the frequency of the activity and how much time it takes them per session.)

How often does this activity take place? OR, “How many days per week/month do you perform this activity?”

How much time does this activity take, each time you perform it?

**MoH salary list tab:**

Discuss with the MoH accountant about the total annual salary (including annualized bonuses and benefits) for each of the staff involved in implementing or supporting AM-related activities at the health facility.

**Medical inputs per child tab:**

Can you tell me on average, what medicines and medical supplies are given to each child admitted to OTP, TSFP and SC during their course of treatment? (Be sure to get the number of units used per child for each item.)

Can you give me a unit cost for each of these items?

(If the facility has a SC center) Can you tell me the approximate cost per bed per day as estimated by the health facility, and the cost of food per day given to each child’s caretaker?

**Community interviews tab:**

(To discuss with any volunteers, CHWs or other relevant community members) We are conducting a costing analysis for the Relapse study by Action Against Hunger. The purpose of the costing analysis is to estimate the average cost of treatment for acutely malnourished children, by both ACF and MoH, in order to estimate the additional cost of repeating treatment for children who have relapsed. The results of the analysis will show how much more it costs to treat a case that receives multiple rounds of treatment due to relapsing, beyond the cost of initial treatment. This information will be used to advocate for efforts to reduce relapse by improving the adherence, coverage, uptake and eventual effectiveness of initial treatment for acute malnutrition.

As part of this, we would like to include an estimate of the value of time contributed by community-level staff. We are looking to find an estimate of the value of these staff people’s time and have a couple of questions.

Would people who work as community health workers or community nutrition volunteers also potentially work in agricultural daily labor?

In your community, what is the daily agricultural wage offered to laborers?

Are there any other daily wage estimates that could be relevant for this kind of staff?
